# Octa­methyldi-μ_3_-oxido-bis­(μ_2_-thio­phene-3-acetato-κ^2^ *O*:*O*′)(thio­phene-3-acetato-κ*O*)tetra­tin(IV)

**DOI:** 10.1107/S1600536809015475

**Published:** 2009-04-30

**Authors:** Muhammad Danish, M. Nawaz Tahir, Nazir Ahmad, Abdul Rauf Raza, Muhammad Ibrahim

**Affiliations:** a Department of Chemistry, University of Sargodha, Sargodha, Pakistan; b Department of Physics, University of Sargodha, Sargodha, Pakistan

## Abstract

In the centrosymmetric title compound, [Sn_4_(CH_3_)_8_(C_6_H_5_O_2_S)_4_O_2_], the central four-membered planar ring (Sn_2_O_2_) makes dihedral angles of 66.28 (12) and 77.43 (11)° with the heterocyclic rings of the bridging and monodentate ligands, respectively. One Sn^IV^ atom adopts a distorted SnO_3_C_2_ trigonal-bipyramidal geometry, with both C atoms in equatorial sites and the other a grossly distorted SnO_4_C_2_ octa­hedral or irregular arrangement. In the crystal, the mol­ecules are connected into pillar-like polymeric units making *R*_2_^2^(12) ring motifs due to inter­molecular C—H⋯O inter­actions. C–H⋯π inter­actions are also present. The O atoms of the chelating ligands and the S atom of the monodentate ligand are disordered over two sets of sites in a 0.65 (6):0.35 (6) ratio

## Related literature

For related structures, see: Danish *et al.* (1995 ▶, 1996 ▶); Ng *et al.* (2001 ▶); Tahir *et al.* (1997*a* ▶,*b* ▶). For graph-set theory, see: Bernstein *et al.* (1995 ▶).
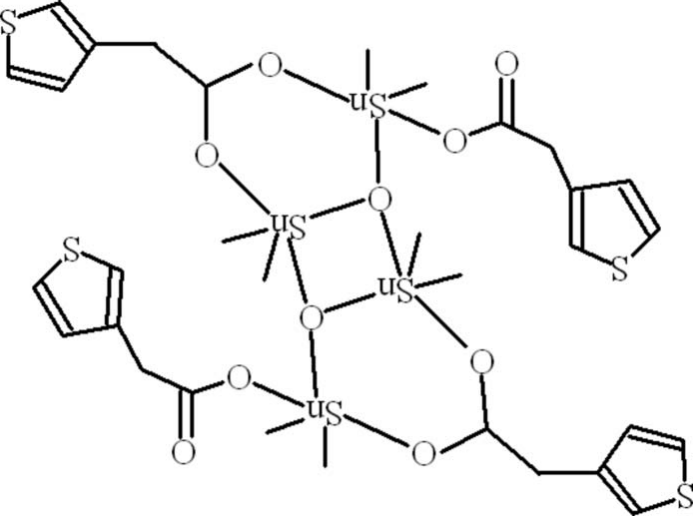


## Experimental

### 

#### Crystal data

[Sn_4_(CH_3_)_8_(C_6_H_5_O_2_S)_4_O_2_]
*M* *_r_* = 1191.79
Triclinic, 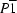

*a* = 9.7330 (5) Å
*b* = 9.7403 (5) Å
*c* = 12.0432 (6) Å
α = 85.407 (2)°
β = 85.259 (1)°
γ = 71.256 (2)°
*V* = 1075.74 (10) Å^3^
*Z* = 1
Mo *K*α radiation
μ = 2.54 mm^−1^
*T* = 296 K
0.20 × 0.15 × 0.13 mm

#### Data collection

Bruker Kappa APEXII CCD diffractometer
Absorption correction: multi-scan (*SADABS*; Bruker, 2005 ▶) *T*_min_ = 0.637, *T*_max_ = 0.719
19310 measured reflections
4012 independent reflections
3441 reflections with *I* > 2σ(*I*)
*R*_int_ = 0.025

#### Refinement

*R*[*F* ^2^ > 2σ(*F* ^2^)] = 0.024
*wR*(*F* ^2^) = 0.059
*S* = 1.04
4012 reflections
250 parameters
H-atom parameters constrained
Δρ_max_ = 0.84 e Å^−3^
Δρ_min_ = −0.65 e Å^−3^

### 

Data collection: *APEX2* (Bruker, 2007 ▶); cell refinement: *SAINT* (Bruker, 2007 ▶); data reduction: *SAINT*; program(s) used to solve structure: *SHELXS97* (Sheldrick, 2008 ▶); program(s) used to refine structure: *SHELXL97* (Sheldrick, 2008 ▶); molecular graphics: *ORTEP-3 for Windows* (Farrugia, 1997 ▶) and *PLATON* (Spek, 2009 ▶); software used to prepare material for publication: *WinGX* (Farrugia, 1999 ▶) and *PLATON*.

## Supplementary Material

Crystal structure: contains datablocks global, I. DOI: 10.1107/S1600536809015475/hb2953sup1.cif

Structure factors: contains datablocks I. DOI: 10.1107/S1600536809015475/hb2953Isup2.hkl

Additional supplementary materials:  crystallographic information; 3D view; checkCIF report

## Figures and Tables

**Table 1:** Selected bond lengths (Å)

Sn1—O1*A*	2.23 (2)
Sn1—O3	2.031 (2)
Sn1—O4	2.207 (3)
Sn1—C7	2.088 (5)
Sn1—C8	2.091 (4)
Sn2—O2*A*	2.312 (17)
Sn2—O3	2.0366 (19)
Sn2—C9	2.106 (5)
Sn2—C10	2.109 (4)
Sn2—O3^i^	2.127 (2)
Sn2—O4^i^	2.670 (3)

Symmetry code: (i) 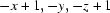.

**Table 2:** Hydrogen-bond geometry (Å, °)

*D*—H⋯*A*	*D*—H	H⋯*A*	*D*⋯*A*	*D*—H⋯*A*
C8—H8*C*⋯O5^ii^	0.96	2.58	3.103 (6)	115
C5—H5⋯CgC^iii^	0.93	2.83	3.513 (5)	131
C10—H10*C*⋯CgC^i^	0.96	2.80	3.697 (5)	156

Symmetry codes: (i) 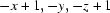; (ii) 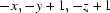; (iii) 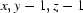. *CgC* is the centriod of the heterocyclic ring (C13–C16/S2*A* or C13–C16/S2*B*).

**Fig. 1.**
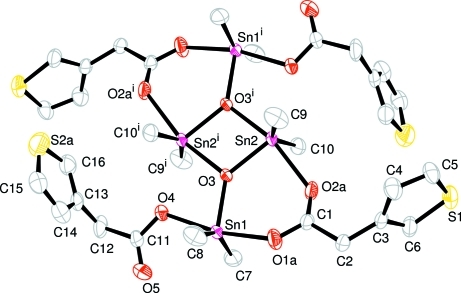
The molecular structure of (I) with displacement ellipsoids drawn at the 30% probability level. H-atoms are not shown for clarity.

## supplementary crystallographic information

### Comment

In continuation to our interest with the tin chemistry in various forms (Danish *et al.*, 1995, 1996), (Tahir *et al.*, 1997*a*, 1997*b*), we report here the title compound (I), (Fig. 1).

The crystal structure of bis(dicyclohexylammonium 3-thienylacetate) (Ng *et al.*, 2001) has been reported which shows disorder in the 3-thienylacetate unit. In our present complex the ligand is also in disorder. The O-atoms of chelating carboxylate are disordered over two sites with occupancy ratio of 0.65:0.35, whereas in other ligands the disorder is present at the S-atoms. In the title molecule symmetry related central four membered ring A (Sn2/O3/Sn2^i^/O3^i^; i = -*x* + 1, -*y*, -*z* + 1) is of course planar. The five membered rings B (C3—C5/S1/C6) and C (C13—C15/S2A/C16) are also planar. The dihedral angles between A/B, A/C and B/C are 66.28 (12)°, 77.43 (11)° and 71.23 (18)°, respectively. Due to intermolecular H-bonding, the stannoxanes are connected in pillar like polymeric form making *R*_2_^2^(12) ring motifs (Bernstein *et al.*, 1995), (Fig. 2). The molecules are also stabilized due to C–H···π interactions (Table 1).

### Experimental

The complex was synthesized by refluxing (CH_3_)_2_SnO (1.66 g, 0.01 mol) and 3-thiopheneacetic acid (1.42 g, 0.01 mol) under argon, in toluene for 4–6 h. Water formed during the reaction was continuously removed by the use of Dean-Stark apparatus. The reaction mixture was brought to room temperature and then boiled with anhydrous activated charcoal and filtered through alumina column. Toluene was removed completely from the filtrate under vacuum. The solid mass thus obtained was purified by repeated crystallization from chloroform-ethanol (8:2) mixture, to obtain colourless prisms of (I).

### Refinement

The H-atoms were positioned geometrically (C—H = 0.93–0.97Å) and refined as riding with U_iso_(H) = 1.2U_eq_(C) or 1.5U_eq_(methyl C).

### Crystal data

**Table:**

[Sn_4_(CH_3_)_8_(C_6_H_5_O_2_S)_4_O_2_]	*Z* = 1
*M**_r_* = 1191.79	*F*(000) = 580
Triclinic, *P*1	*D*_x_ = 1.840 Mg m^−^^3^
Hall symbol: -P 1	Mo *K*α radiation, λ = 0.71073 Å
*a* = 9.7330 (5) Å	Cell parameters from 3441 reflections
*b* = 9.7403 (5) Å	θ = 2.2–25.5°
*c* = 12.0432 (6) Å	µ = 2.54 mm^−^^1^
α = 85.407 (2)°	*T* = 296 K
β = 85.259 (1)°	Prism, colourless
γ = 71.256 (2)°	0.20 × 0.15 × 0.13 mm
*V* = 1075.74 (10) Å^3^	

### Data collection

**Table:**

Bruker Kappa APEXII CCD diffractometer	4012 independent reflections
Radiation source: fine-focus sealed tube	3441 reflections with *I* > 2σ(*I*)
graphite	*R*_int_ = 0.025
Detector resolution: 7.80 pixels mm^-1^	θ_max_ = 25.5°, θ_min_ = 2.2°
ω scans	*h* = −11→11
Absorption correction: multi-scan (*SADABS*; Bruker, 2005)	*k* = −11→11
*T*_min_ = 0.637, *T*_max_ = 0.719	*l* = −14→14
19310 measured reflections	

### Refinement

**Table:**

Refinement on *F*^2^	Secondary atom site location: difference Fourier map
Least-squares matrix: full	Hydrogen site location: inferred from neighbouring sites
*R*[*F*^2^ > 2σ(*F*^2^)] = 0.024	H-atom parameters constrained
*wR*(*F*^2^) = 0.059	*w* = 1/[σ^2^(*F*_o_^2^) + (0.0219*P*)^2^ + 1.4193*P*] where *P* = (*F*_o_^2^ + 2*F*_c_^2^)/3
*S* = 1.03	(Δ/σ)_max_ = 0.001
4012 reflections	Δρ_max_ = 0.84 e Å^−^^3^
250 parameters	Δρ_min_ = −0.65 e Å^−^^3^
0 restraints	Extinction correction: *SHELXL97* (Sheldrick, 2008), Fc^*^=kFc[1+0.001xFc^2^λ^3^/sin(2θ)]^-1/4^
Primary atom site location: structure-invariant direct methods	Extinction coefficient: 0.00156 (19)

### Special details

**Table:**

Geometry. Bond distances, angles *etc*. have been calculated using the rounded fractional coordinates. All su's are estimated from the variances of the (full) variance-covariance matrix. The cell e.s.d.'s are taken into account in the estimation of distances, angles and torsion angles
Refinement. Refinement of *F*^2^ against ALL reflections. The weighted *R*-factor *wR* and goodness of fit *S* are based on *F*^2^, conventional *R*-factors *R* are based on *F*, with *F* set to zero for negative *F*^2^. The threshold expression of *F*^2^ > σ(*F*^2^) is used only for calculating *R*-factors(gt) *etc*. and is not relevant to the choice of reflections for refinement. *R*-factors based on *F*^2^ are statistically about twice as large as those based on *F*, and *R*- factors based on ALL data will be even larger.

### Fractional atomic coordinates and isotropic or equivalent isotropic displacement parameters (Å^2^)

**Table:**

	*x*	*y*	*z*	*U*_iso_*/*U*_eq_	Occ. (<1)
Sn1	0.18885 (2)	0.25808 (2)	0.45386 (2)	0.0444 (1)	
Sn2	0.48666 (2)	−0.09190 (2)	0.39587 (2)	0.0446 (1)	
S1	0.27895 (18)	−0.25877 (16)	−0.05837 (11)	0.0934 (5)	
S2A	0.3409 (3)	0.25077 (19)	0.99888 (15)	0.1310 (8)	0.635
O1A	0.1177 (13)	0.137 (3)	0.333 (2)	0.076 (4)	0.65 (6)
O2A	0.3179 (12)	−0.023 (3)	0.2610 (14)	0.065 (4)	0.65 (6)
O3	0.3779 (2)	0.0937 (2)	0.47382 (18)	0.0410 (7)	
O4	0.2813 (3)	0.3298 (3)	0.5913 (2)	0.0584 (9)	
O5	0.0925 (4)	0.5232 (4)	0.5897 (3)	0.1044 (16)	
C1	0.1856 (4)	0.0434 (4)	0.2645 (3)	0.0497 (12)	
C2	0.0913 (4)	0.0161 (4)	0.1808 (3)	0.0562 (12)	
C3	0.1613 (4)	−0.1092 (4)	0.1087 (3)	0.0532 (12)	
C4	0.1850 (5)	−0.2556 (5)	0.1418 (4)	0.0771 (19)	
C5	0.2468 (5)	−0.3522 (5)	0.0595 (4)	0.0719 (16)	
C6	0.2094 (6)	−0.0968 (5)	0.0020 (4)	0.0756 (19)	
C7	0.2415 (5)	0.4034 (5)	0.3342 (4)	0.0789 (17)	
C8	0.0351 (4)	0.1981 (5)	0.5611 (4)	0.0822 (18)	
C9	0.3936 (5)	−0.2493 (5)	0.4656 (5)	0.089 (2)	
C10	0.6134 (5)	−0.0291 (5)	0.2633 (3)	0.0712 (16)	
C11	0.2064 (5)	0.4535 (4)	0.6276 (3)	0.0628 (14)	
C12	0.2698 (6)	0.5070 (5)	0.7194 (4)	0.0832 (19)	
C13	0.2700 (5)	0.4165 (4)	0.8259 (3)	0.0607 (14)	
C14	0.1451 (6)	0.4020 (6)	0.8834 (4)	0.0840 (19)	
C15	0.1689 (6)	0.3121 (5)	0.9827 (4)	0.0761 (19)	
C16	0.3894 (5)	0.3378 (6)	0.8800 (4)	0.0839 (19)	
S2B	0.3409 (3)	0.25077 (19)	0.99888 (15)	0.1310 (8)	0.365
O2B	0.283 (5)	−0.0624 (18)	0.299 (4)	0.065 (7)	0.35 (6)
O1B	0.156 (5)	0.168 (2)	0.296 (3)	0.062 (7)	0.35 (6)
H2A	0.00668	0.00014	0.22101	0.0672*	
H4	0.16091	−0.28575	0.21394	0.0921*	
H5	0.26718	−0.45241	0.06791	0.0864*	
H7A	0.19235	0.40649	0.26765	0.1183*	
H7B	0.34458	0.37212	0.31712	0.1183*	
H6A	0.20504	−0.00826	−0.03533	0.0906*	
H2B	0.05705	0.10329	0.13256	0.0672*	
H8B	0.05988	0.09419	0.56672	0.1230*	
H8C	−0.05934	0.23970	0.53209	0.1230*	
H9A	0.30262	−0.20216	0.50480	0.1329*	
H9B	0.45826	−0.31449	0.51661	0.1329*	
H9C	0.37745	−0.30285	0.40721	0.1329*	
H10A	0.67382	0.01893	0.29210	0.1063*	
H10B	0.55061	0.03594	0.21106	0.1063*	
H10C	0.67335	−0.11358	0.22649	0.1063*	
H12A	0.36857	0.50390	0.69644	0.0997*	
H12B	0.21327	0.60710	0.73188	0.0997*	
H14	0.05271	0.44812	0.85838	0.1013*	
H15	0.09708	0.29176	1.03071	0.0912*	
H16A	0.48440	0.33181	0.85601	0.1007*	
H7C	0.21167	0.49849	0.36272	0.1183*	
H8A	0.03409	0.23280	0.63361	0.1230*	

### Atomic displacement parameters (Å^2^)

**Table:**

	*U*^11^	*U*^22^	*U*^33^	*U*^12^	*U*^13^	*U*^23^
Sn1	0.0437 (1)	0.0386 (1)	0.0463 (2)	−0.0034 (1)	−0.0056 (1)	−0.0128 (1)
Sn2	0.0470 (1)	0.0397 (1)	0.0452 (2)	−0.0066 (1)	−0.0063 (1)	−0.0177 (1)
S1	0.1234 (11)	0.0908 (9)	0.0681 (8)	−0.0294 (8)	−0.0098 (7)	−0.0323 (7)
S2A	0.206 (2)	0.0932 (11)	0.0868 (11)	−0.0321 (12)	−0.0320 (12)	−0.0049 (9)
O1A	0.058 (4)	0.088 (8)	0.084 (8)	−0.016 (4)	−0.007 (4)	−0.050 (7)
O2A	0.054 (4)	0.091 (10)	0.051 (5)	−0.015 (4)	−0.010 (3)	−0.032 (5)
O3	0.0391 (11)	0.0368 (12)	0.0424 (12)	−0.0025 (9)	−0.0042 (9)	−0.0133 (9)
O4	0.0644 (16)	0.0503 (14)	0.0575 (16)	−0.0070 (12)	−0.0136 (12)	−0.0216 (12)
O5	0.099 (3)	0.078 (2)	0.108 (3)	0.0243 (19)	−0.031 (2)	−0.036 (2)
C1	0.056 (2)	0.051 (2)	0.047 (2)	−0.0207 (18)	−0.0110 (17)	−0.0073 (17)
C2	0.063 (2)	0.057 (2)	0.052 (2)	−0.0193 (18)	−0.0162 (18)	−0.0092 (17)
C3	0.061 (2)	0.057 (2)	0.049 (2)	−0.0237 (18)	−0.0167 (17)	−0.0112 (17)
C4	0.103 (4)	0.065 (3)	0.073 (3)	−0.041 (3)	0.002 (3)	−0.008 (2)
C5	0.095 (3)	0.052 (2)	0.077 (3)	−0.033 (2)	0.002 (2)	−0.019 (2)
C6	0.116 (4)	0.063 (3)	0.052 (3)	−0.030 (3)	−0.016 (2)	−0.010 (2)
C7	0.099 (3)	0.062 (3)	0.059 (3)	−0.006 (2)	0.000 (2)	0.008 (2)
C8	0.051 (2)	0.075 (3)	0.117 (4)	−0.019 (2)	0.014 (2)	−0.008 (3)
C9	0.066 (3)	0.067 (3)	0.143 (5)	−0.033 (2)	−0.002 (3)	−0.016 (3)
C10	0.088 (3)	0.065 (3)	0.047 (2)	−0.008 (2)	0.007 (2)	−0.0032 (19)
C11	0.083 (3)	0.049 (2)	0.054 (2)	−0.013 (2)	−0.009 (2)	−0.0174 (18)
C12	0.137 (4)	0.069 (3)	0.058 (3)	−0.047 (3)	−0.012 (3)	−0.021 (2)
C13	0.077 (3)	0.059 (2)	0.052 (2)	−0.024 (2)	−0.005 (2)	−0.0255 (19)
C14	0.076 (3)	0.085 (3)	0.095 (4)	−0.028 (3)	0.009 (3)	−0.032 (3)
C15	0.095 (4)	0.067 (3)	0.072 (3)	−0.037 (3)	0.017 (3)	−0.013 (2)
C16	0.076 (3)	0.098 (4)	0.079 (3)	−0.022 (3)	−0.008 (2)	−0.032 (3)
S2B	0.206 (2)	0.0932 (11)	0.0868 (11)	−0.0321 (12)	−0.0320 (12)	−0.0049 (9)
O2B	0.075 (12)	0.052 (7)	0.076 (15)	−0.021 (5)	−0.033 (12)	−0.012 (7)
O1B	0.076 (15)	0.047 (7)	0.062 (11)	−0.011 (6)	−0.025 (9)	−0.021 (6)

### Geometric parameters (Å, °)

**Table:**

Sn1—O1A	2.23 (2)	C3—C6	1.341 (6)
Sn1—O3	2.031 (2)	C4—C5	1.384 (7)
Sn1—O4	2.207 (3)	C11—C12	1.512 (7)
Sn1—C7	2.088 (5)	C12—C13	1.497 (6)
Sn1—C8	2.091 (4)	C13—C16	1.355 (7)
Sn1—O1B	2.24 (4)	C13—C14	1.390 (8)
Sn2—O2A	2.312 (17)	C14—C15	1.414 (7)
Sn2—O3	2.0366 (19)	C2—H2A	0.9700
Sn2—C9	2.106 (5)	C2—H2B	0.9700
Sn2—C10	2.109 (4)	C4—H4	0.9300
Sn2—O3^i^	2.127 (2)	C5—H5	0.9300
Sn2—O4^i^	2.670 (3)	C6—H6A	0.9300
Sn2—O2B	2.31 (5)	C7—H7A	0.9600
Sn2—Sn2^i^	3.2694 (4)	C7—H7B	0.9600
S1—C5	1.686 (5)	C7—H7C	0.9600
S1—C6	1.701 (5)	C8—H8A	0.9600
S2A—C15	1.609 (7)	C8—H8B	0.9600
S2A—C16	1.715 (5)	C8—H8C	0.9600
S2B—C16	1.715 (5)	C9—H9A	0.9600
S2B—C15	1.609 (7)	C9—H9B	0.9600
O1A—C1	1.26 (3)	C9—H9C	0.9600
O1B—C1	1.24 (2)	C10—H10A	0.9600
O2A—C1	1.240 (19)	C10—H10B	0.9600
O2B—C1	1.23 (3)	C10—H10C	0.9600
O4—C11	1.281 (5)	C12—H12A	0.9700
O5—C11	1.204 (6)	C12—H12B	0.9700
C1—C2	1.511 (5)	C14—H14	0.9300
C2—C3	1.498 (5)	C15—H15	0.9300
C3—C4	1.399 (6)	C16—H16A	0.9300
			
O1A—Sn1—O3	91.9 (6)	C3—C4—C5	115.2 (4)
O1A—Sn1—O4	167.3 (7)	S1—C5—C4	109.0 (3)
O1A—Sn1—C7	95.5 (6)	S1—C6—C3	113.4 (3)
O1A—Sn1—C8	82.7 (5)	O4—C11—C12	116.3 (4)
O3—Sn1—O4	77.69 (9)	O4—C11—O5	121.5 (4)
O3—Sn1—C7	104.94 (15)	O5—C11—C12	122.2 (4)
O3—Sn1—C8	104.92 (14)	C11—C12—C13	111.4 (4)
O1B—Sn1—O3	90.8 (9)	C12—C13—C14	124.0 (5)
O4—Sn1—C7	94.25 (15)	C12—C13—C16	125.7 (5)
O4—Sn1—C8	92.93 (15)	C14—C13—C16	110.4 (4)
O1B—Sn1—O4	164.9 (12)	C13—C14—C15	115.1 (5)
C7—Sn1—C8	150.12 (18)	S2A—C15—C14	108.6 (4)
O1B—Sn1—C7	79.1 (9)	S2B—C15—C14	108.6 (4)
O1B—Sn1—C8	99.6 (11)	S2A—C16—C13	110.5 (4)
O2A—Sn2—O3	89.1 (6)	S2B—C16—C13	110.5 (4)
O2A—Sn2—C9	90.5 (6)	C1—C2—H2A	108.00
O2A—Sn2—C10	80.5 (4)	C1—C2—H2B	108.00
Sn2^i^—Sn2—O2A	128.1 (6)	C3—C2—H2A	108.00
O2A—Sn2—O3^i^	164.4 (7)	C3—C2—H2B	108.00
O2A—Sn2—O4^i^	128.2 (6)	H2A—C2—H2B	107.00
O3—Sn2—C9	105.78 (15)	C3—C4—H4	122.00
O3—Sn2—C10	105.39 (14)	C5—C4—H4	122.00
O2B—Sn2—O3	89.1 (7)	S1—C5—H5	126.00
Sn2^i^—Sn2—O3	39.25 (6)	C4—C5—H5	125.00
O3—Sn2—O3^i^	76.53 (8)	S1—C6—H6A	123.00
O3—Sn2—O4^i^	142.67 (8)	C3—C6—H6A	123.00
C9—Sn2—C10	147.33 (19)	Sn1—C7—H7A	109.00
O2B—Sn2—C9	73.5 (9)	Sn1—C7—H7B	110.00
Sn2^i^—Sn2—C9	105.84 (16)	Sn1—C7—H7C	109.00
O3^i^—Sn2—C9	99.16 (16)	H7A—C7—H7B	109.00
O4^i^—Sn2—C9	78.01 (15)	H7A—C7—H7C	109.00
O2B—Sn2—C10	97.5 (12)	H7B—C7—H7C	109.00
Sn2^i^—Sn2—C10	104.38 (12)	Sn1—C8—H8A	109.00
O3^i^—Sn2—C10	97.34 (14)	Sn1—C8—H8B	109.00
O4^i^—Sn2—C10	83.18 (14)	Sn1—C8—H8C	109.00
Sn2^i^—Sn2—O2B	127.4 (8)	H8A—C8—H8B	110.00
O2B—Sn2—O3^i^	161.6 (11)	H8A—C8—H8C	109.00
O2B—Sn2—O4^i^	126.4 (5)	H8B—C8—H8C	109.00
Sn2^i^—Sn2—O3^i^	37.29 (5)	Sn2—C9—H9A	109.00
Sn2^i^—Sn2—O4^i^	103.52 (6)	Sn2—C9—H9B	109.00
O3^i^—Sn2—O4^i^	66.28 (8)	Sn2—C9—H9C	109.00
C5—S1—C6	92.5 (2)	H9A—C9—H9B	109.00
C15—S2A—C16	95.4 (3)	H9A—C9—H9C	109.00
C15—S2B—C16	95.4 (3)	H9B—C9—H9C	110.00
Sn1—O1A—C1	133.2 (11)	Sn2—C10—H10A	109.00
Sn1—O1B—C1	134 (2)	Sn2—C10—H10B	109.00
Sn2—O2A—C1	132.8 (12)	Sn2—C10—H10C	109.00
Sn2—O2B—C1	134 (2)	H10A—C10—H10B	109.00
Sn2—O3—Sn2^i^	103.47 (9)	H10A—C10—H10C	110.00
Sn1—O3—Sn2^i^	120.70 (10)	H10B—C10—H10C	110.00
Sn1—O3—Sn2	135.83 (11)	C11—C12—H12A	109.00
Sn2^i^—O4—C11	149.5 (3)	C11—C12—H12B	109.00
Sn1—O4—Sn2^i^	95.14 (9)	C13—C12—H12A	109.00
Sn1—O4—C11	115.4 (3)	C13—C12—H12B	109.00
O1A—C1—O2A	125.9 (13)	H12A—C12—H12B	108.00
O1A—C1—C2	114.1 (9)	C13—C14—H14	122.00
O2A—C1—C2	120.0 (10)	C15—C14—H14	122.00
O1B—C1—O2B	125 (3)	S2A—C15—H15	126.00
O1B—C1—C2	118 (2)	C14—C15—H15	126.00
O2B—C1—C2	116.6 (18)	S2B—C15—H15	126.00
C1—C2—C3	116.3 (3)	S2A—C16—H16A	125.00
C2—C3—C4	125.4 (4)	C13—C16—H16A	125.00
C2—C3—C6	124.6 (4)	S2B—C16—H16A	125.00
C4—C3—C6	110.0 (4)		
			
O3—Sn1—O1A—C1	−29 (2)	C10—Sn2—Sn2^i^—O2A^i^	−90.9 (6)
C7—Sn1—O1A—C1	76 (2)	C10—Sn2—Sn2^i^—O3^i^	−83.16 (16)
C8—Sn1—O1A—C1	−134 (2)	C10—Sn2—Sn2^i^—C9^i^	12.55 (19)
O1A—Sn1—O3—Sn2	11.1 (6)	C10—Sn2—Sn2^i^—C10^i^	180.0 (2)
O1A—Sn1—O3—Sn2^i^	−168.5 (6)	O3^i^—Sn2—Sn2^i^—O3	180.00 (14)
O4—Sn1—O3—Sn2	−176.29 (17)	O3^i^—Sn2—Sn2^i^—O4	176.85 (11)
O4—Sn1—O3—Sn2^i^	4.13 (11)	O4^i^—Sn2—Sn2^i^—O3	−176.85 (11)
C7—Sn1—O3—Sn2	−85.1 (2)	O4^i^—Sn2—Sn2^i^—O4	180.00 (8)
C7—Sn1—O3—Sn2^i^	95.29 (17)	O3—Sn2—O3^i^—Sn1^i^	−179.70 (13)
C8—Sn1—O3—Sn2	94.0 (2)	O3—Sn2—O3^i^—Sn2^i^	0.00 (10)
C8—Sn1—O3—Sn2^i^	−85.63 (17)	C9—Sn2—O3^i^—Sn1^i^	76.14 (17)
O3—Sn1—O4—C11	178.3 (3)	C9—Sn2—O3^i^—Sn2^i^	−104.16 (16)
O3—Sn1—O4—Sn2^i^	−2.84 (8)	C10—Sn2—O3^i^—Sn1^i^	−75.56 (16)
C7—Sn1—O4—C11	73.9 (3)	C10—Sn2—O3^i^—Sn2^i^	104.14 (15)
C7—Sn1—O4—Sn2^i^	−107.23 (15)	O2A—Sn2—O4^i^—Sn1^i^	170.4 (6)
C8—Sn1—O4—C11	−77.1 (3)	O2A—Sn2—O4^i^—C11^i^	−7.7 (8)
C8—Sn1—O4—Sn2^i^	101.80 (14)	O3—Sn2—O4^i^—Sn1^i^	−8.27 (18)
O3—Sn2—O2A—C1	−35 (2)	O3—Sn2—O4^i^—C11^i^	173.7 (5)
C9—Sn2—O2A—C1	71 (2)	C9—Sn2—O4^i^—Sn1^i^	−108.63 (18)
C10—Sn2—O2A—C1	−140 (2)	C9—Sn2—O4^i^—C11^i^	73.3 (5)
Sn2^i^—Sn2—O2A—C1	−39 (2)	C10—Sn2—O4^i^—Sn1^i^	98.23 (15)
O4^i^—Sn2—O2A—C1	146.3 (19)	C10—Sn2—O4^i^—C11^i^	−79.9 (5)
O2A—Sn2—O3—Sn1	6.5 (5)	C6—S1—C5—C4	−0.7 (4)
O2A—Sn2—O3—Sn2^i^	−173.9 (5)	C5—S1—C6—C3	−0.3 (5)
C9—Sn2—O3—Sn1	−83.8 (2)	C16—S2A—C15—C14	0.2 (4)
C9—Sn2—O3—Sn2^i^	95.88 (17)	C15—S2A—C16—C13	0.0 (4)
C10—Sn2—O3—Sn1	86.34 (19)	Sn1—O1A—C1—O2A	13 (3)
C10—Sn2—O3—Sn2^i^	−94.03 (15)	Sn1—O1A—C1—C2	−166.7 (15)
Sn2^i^—Sn2—O3—Sn1	−179.6 (2)	Sn2—O2A—C1—O1A	29 (3)
O3^i^—Sn2—O3—Sn1	−179.63 (17)	Sn2—O2A—C1—C2	−152.2 (14)
O3^i^—Sn2—O3—Sn2^i^	0.00 (8)	Sn1—O4—C11—O5	1.2 (5)
O4^i^—Sn2—O3—Sn1	−174.57 (12)	Sn1—O4—C11—C12	−178.2 (3)
O4^i^—Sn2—O3—Sn2^i^	5.06 (18)	Sn2^i^—O4—C11—O5	−176.6 (3)
O2A—Sn2—Sn2^i^—O3	7.8 (6)	Sn2^i^—O4—C11—C12	4.0 (7)
O2A—Sn2—Sn2^i^—O4	4.6 (6)	O1A—C1—C2—C3	−171.2 (13)
O2A—Sn2—Sn2^i^—O2A^i^	−180.0 (8)	O2A—C1—C2—C3	9.5 (13)
O2A—Sn2—Sn2^i^—O3^i^	−172.2 (6)	C1—C2—C3—C4	78.1 (5)
O2A—Sn2—Sn2^i^—C9^i^	−76.5 (6)	C1—C2—C3—C6	−102.5 (5)
O2A—Sn2—Sn2^i^—C10^i^	90.9 (6)	C2—C3—C4—C5	177.7 (4)
O3—Sn2—Sn2^i^—O4	−3.15 (11)	C6—C3—C4—C5	−1.7 (6)
O3—Sn2—Sn2^i^—O2A^i^	172.2 (6)	C2—C3—C6—S1	−178.3 (3)
O3—Sn2—Sn2^i^—O3^i^	−180.00 (14)	C4—C3—C6—S1	1.2 (6)
O3—Sn2—Sn2^i^—C9^i^	−84.29 (17)	C3—C4—C5—S1	1.5 (6)
O3—Sn2—Sn2^i^—C10^i^	83.16 (16)	O4—C11—C12—C13	−68.4 (5)
C9—Sn2—Sn2^i^—O3	−95.71 (17)	O5—C11—C12—C13	112.2 (5)
C9—Sn2—Sn2^i^—O4	−98.86 (15)	C11—C12—C13—C14	−61.4 (6)
C9—Sn2—Sn2^i^—O2A^i^	76.5 (6)	C11—C12—C13—C16	118.7 (5)
C9—Sn2—Sn2^i^—O3^i^	84.29 (17)	C12—C13—C14—C15	−179.6 (4)
C9—Sn2—Sn2^i^—C9^i^	−180.0 (2)	C16—C13—C14—C15	0.3 (6)
C9—Sn2—Sn2^i^—C10^i^	−12.55 (19)	C12—C13—C16—S2A	179.8 (4)
C10—Sn2—Sn2^i^—O3	96.85 (16)	C14—C13—C16—S2A	−0.2 (5)
C10—Sn2—Sn2^i^—O4	93.70 (15)	C13—C14—C15—S2A	−0.4 (6)

Symmetry codes: (i) −*x*+1, −*y*, −*z*+1.

### Hydrogen-bond geometry (Å, °)

**Table:**

*D*—H···*A*	*D*—H	H···*A*	*D*···*A*	*D*—H···*A*
C8—H8C···O5^ii^	0.96	2.58	3.103 (6)	115
C5—H5···CgC^iii^	0.93	2.83	3.513 (5)	131
C10—H10C···CgC^i^	0.96	2.80	3.697 (5)	156

Symmetry codes: (ii) −*x*, −*y*+1, −*z*+1; (iii) *x*, *y*−1, *z*−1; (i) −*x*+1, −*y*, −*z*+1.
